# LncRNA *SNHGs*调控非小细胞肺癌的生物学行为研究进展

**DOI:** 10.3779/j.issn.1009-3419.2023.102.41

**Published:** 2023-11-20

**Authors:** Xincen CAO, Liang CHEN

**Affiliations:** 210000 南京，南京医科大学第一附属医院胸外科; Department of Thoracic Surgery, First Affiliated Hospital of Nanjing Medical University, Nanjing 210000, China

**Keywords:** 长链非编码RNA, 小核仁RNA宿主基因, 肺肿瘤, Long non-coding RNA, Small nucleolar RNA host gene, Lung neoplasms

## Abstract

肺癌是我国发病率和死亡率最高的恶性肿瘤之一，其发生发展机制及治疗手段是当前研究的热点。近年来，针对各种肿瘤驱动基因的分子靶向药物的出现显著提高了肺癌患者的生存率及生活质量，掀起了一波研究新型治疗靶标的热潮。其中长链非编码RNA（long non-coding RNA, lncRNA）在肿瘤的恶性行为中发挥了至关重要的作用，引起了人们的广泛关注。研究表明，lncRNA小核仁RNA宿主基因（small nucleolar RNA host gene, SNHG）家族部分成员在非小细胞肺癌（non-small cell lung cancer, NSCLC）等多种肿瘤组织中异常表达，参与肿瘤细胞的增殖、侵袭和转移等生物学过程，可能是NSCLC的诊断和预后的生物标志物，以及潜在的治疗新靶点。在这篇综述中，我们总结并探讨了SNHGs在NSCLC中的最新研究进展，以期为NSCLC的诊断及治疗提供新思路。

## Abstract (en)

Lung cancer is one of the malignant tumors with the highest incidence and mortality rate in China, and its occurrence and development mechanism and treatment methods are the current research focuses. In recent years, the emergence of drugs targeting various tumor driver genes has significantly improved patients’ survival and quality of life, setting off a wave of research on new therapeutic targets. Among them, long non-coding RNA (lncRNA) plays a crucial role in the malignant behavior of tumors, which has attracted widespread attention. Shown by a large number of studies, partial members of lncRNA small nucleolar RNA host gene (SNHG) family are aberrantly expressed in many maliglant tumors including non-small cell lung cancer (NSCLC) and participate in cell proliferation, invasion and migration, which may act as a new diagnostic and prognostic biomarker and can be a therapeutic target of NSCLC. In this review, we comprehensively summarize and explore the recent investigation of SNHGs in NSCLC in order to provide new ideas for the diagnosis and treatment of NSCLC.

肺癌是全球最常见的恶性肿瘤之一^[1]^。根据其病理学特征，肺癌分为小细胞肺癌和非小细胞肺癌（non-small cell lung cancer, NSCLC），其中NSCLC占肺癌发病率的85%^[2]^，又可细分为腺癌、大细胞癌和鳞状细胞癌^[3,4]^。尽管近年来，肺癌的靶向和免疫治疗已经取得了很大进展，但晚期NSCLC患者的预后仍然较差，5年生存率低于20%^[5,6]^。因此，进一步了解NSCLC内在的分子调控机制，识别新的生物标志物和更有效的治疗靶点，对于改善患者的远期预后意义深远。

长链非编码RNA（long non-coding RNA, lncRNA）是一类转录量超过200个核苷酸、编码能力有限或无编码能力的非编码RNA（non-coding RNA, ncRNA）^[7]^。过去，人们认为lncRNA不具备任何生物学功能，是基因组转录的噪音。然而，越来越多的证据^[8]^表明，lncRNA在X染色体印迹、干细胞分化、免疫应答、癌细胞增殖和化疗耐药等细胞生物学过程中发挥着重大作用。

小核仁RNA宿主基因（small nucleolar RNA host gene, SNHG）家族成员是一类新发现的lncRNA，已被证实在多种肿瘤中异常表达，与肿瘤的进展密切相关。本综述将结合国内外文献，概述lncRNA SNHGs在NSCLC中的作用及机制，为寻找NSCLC的诊疗方法提供新的切入点，以期提高临床患者的长期生存率。

## 1 LncRNA SNHGs概述

LncRNA SNHGs是小核仁RNA（small nucleolar RNA, snoRNA）的宿主基因。snoRNA是一类长度为60-300 nt的ncRNA，主要富集于核仁，且代谢稳定，由lncRNA SNHGs内含子中的RNA聚合酶II转录而来。snoRNA不仅参与了核糖体RNA前体的剪接加工，还能够指导核内小RNA（small nuclear RNA, snRNA）、转运RNA（transfer RNA, tRNA）和信使RNA（messenger RNA, mRNA）的转录后修饰，并与核糖体生物合成密切相关^[9,10]^。此外，还有相当数量的snoRNA功能不明，被称为孤儿snoRNA。

SNHG家族中有22个成员（SNHG1-SNHG22）。它们已被证实在各种肿瘤的发生发展中发挥了重要作用，如肺癌^[11]^、肝癌^[12]^、甲状腺癌^[13]^、乳腺癌^[14]^、卵巢癌^[15]^、胰腺癌^[16]^等。

研究^[17]^表明，lncRNA SNHGs主要通过五种分子作用机制参与癌症调节：（1）影响DNA甲基化；（2）与转录因子相互作用；（3）充当竞争性内源RNA（competitive endogenous RNA, ceRNA）；（4）直接结合mRNA并抑制蛋白翻译；（5）与蛋白质相互作用阻碍蛋白质的泛素化。区别于其他lncRNA，SNHGs的作用机制取决于它们的细胞定位。如在细胞核中，SNHGs主要通过甲基化酶影响DNA甲基化或与转录因子相互作用调控基因转录；而在细胞质中，SNHGs可以作为ceRNA来影响mRNA的稳定性和蛋白质的生物活性。

## 2 LncRNA SNHGs与NSCLC的关系

### 2.1 LncRNA

SNHGs在NSCLC中的生物学作用 LncRNA SNHGs在NSCLC中特异性表达，并能对多种肿瘤生物学过程进行调控。早在2014年，You等^[18]^通过定量逆转录聚合酶链反应（quantitative reverse‐transcription polymerase chain reaction, qRT-PCR）检测出SNHG1在肺癌细胞系中高表达。研究^[11]^表明，SNHG1可以增强Wnt/β-catenin信号传导的活性，促进NSCLC细胞的增殖、侵袭和转移能力。Nie等^[19]^在研究人乳头瘤病毒（human papillomavirus, HPV）时发现SNHG1在感染HPV16 E6的NSCLC细胞中上调，且SNHG1通过激活核因子κB（nuclear factor kappa B, NF-κB）信号通路及信号传导子和转录活化子1（signal transducers and activators of transcription 1, STAT1）通路，释放血管内皮生长因子D，促进人脐静脉内皮细胞的血管形成。GAS5又被称为SNHG2，Shen所在团队^[20]^构建了GAS5的过表达体系，通过CCK8、Edu、Transwell等体外功能实验证明GAS5抑制了肿瘤细胞增殖、凋亡和侵袭的能力。此外，GAS5通过调节E-钙黏蛋白（E-cadherin）和N-钙黏蛋白（N-cadherin）的表达，可以逆转NSCLC的上皮-间充质转化（epithelial-mesenchymal transition, EMT）过程，降低了细胞的活力，延缓了肿瘤的转移及侵袭^[21]^。另有研究^[22]^表明，脂肪含量和肥胖相关蛋白（fat mass and obesity-associated protein, FTO）介导的GAS5通过与上移蛋白1（up-frameshift protein 1, UPF1）相互作用降低了mRNA含溴结构域蛋白4（bromodomain-containing protein 4, BRD4）的稳定性，促进了细胞的凋亡及自噬。有研究^[23]^发现，SNHG4可以充当miR-let-7e的分子海绵，并靶向作用于赖氨酸脱甲基酶3A（lysine demethylase 3A, KDM3A），而KDM3A可以降低p21的转录活性，促进NSCLC细胞的增殖侵袭能力及周期进展，同时抑制细胞凋亡。Wang所在团队^[24]^发现，SNHG6通过将zeste同源物增强子2（enhancer of zeste homolog 2, EZH2）募集到p27的启动子区域并增加H3K27me3的富集，从而在表观遗传学上降低p27的表达，抑制细胞周期的G_1_/S期转换，并促进NSCLC的肿瘤发展。随后一项研究^[25]^发现，SNHG6的沉默可以调节p21的表达来抑制肿瘤细胞的增殖并促进细胞凋亡。2018年，Chen等^[26]^分析了120例NSCLC患者的组织样本，发现SNHG8在NSCLC中过表达。敲低SNHG8通过靶向作用于miR-542-3p/CCND1（cyclin D1）/细胞周期蛋白依赖性激酶6（cyclin dependent kinase 6, CDK6）轴，使细胞周期阻滞在G_0_/G_1_期，并激活Caspase-3诱导细胞凋亡，进而抑制NSCLC细胞的体外和体内增殖能力。Huang所在团队^[27]^探究了SNHG12在NSCLC免疫逃逸中的分子机制，他们发现沉默SNHG12会导致NSCLC细胞的增殖能力降低并诱导细胞凋亡，同时促进了外周血单核细胞（peripheral blood mononuclear cell, PBMC）的增殖和CD8^+^ T细胞的比例，减少了NSCLC的免疫逃逸。DANCR又名SNHG13，Guo等的研究^[28]^表明，沉默DANCR通过抑制EZH2介导的p21启动子沉默，增加了p21表达，促进了肿瘤细胞凋亡并阻滞了细胞周期，进而抑制了NSCLC细胞增殖。另有研究^[29]^发现，SNHG15的沉默可以海绵化miR-486并作用于CDK14，使细胞周期阻滞于G_0_/G_1_期，从而抑制肿瘤的增殖，促进细胞凋亡。Han等的研究^[30]^表明，SNHG16通过靶向作用于miR-146a而促进肿瘤进展，且二者可以共同调控黏蛋白5AC（mucin 5AC, MUC5AC）发挥功能。而MUC5AC是一种与NSCLC术后转移和复发相关的人呼吸道主要黏蛋白，在促进细胞增殖、迁移和侵袭方面具有积极作用。

总之，lncRNA SNHGs参与了NSCLC多种生物学过程，包括肿瘤细胞的增殖、自噬、迁移、侵袭、凋亡，细胞周期的调控，肿瘤血管的形成及肿瘤的免疫逃逸等等。其中，SNHG1、SNHG3、SNHG4、SNHG6、SNHG8、SNHG11、SNHG12、DANCR、SNHG14、SNHG15、SNHG16、SNHG17、SNHG18、SNHG20作为癌基因促进肿瘤生长，而GAS5和SNHG10则发挥肿瘤抑制基因的作用。然而，关于SNHG5、SNHG7、SNHG9在NSCLC中发挥促癌还是抑癌效应，部分学者的研究仍存在分歧，需要更多的证据佐证。此外，SNHG19、SNHG21、SNHG22在NSCLC中尚未有相关的研究报道（表1）。

**表1:** SNHG家族成员特征

SNHG member	Chromosomal location	GENE ID	Role in non-small cell lung cancer
SNHG1	11q12.3	23642	Oncogene
GAS5	1q25.1	60674	Antioncogene
SNHG3	1p35.3	8420	Oncogene
SNHG4	5q31.2	724102	Oncogene
SNHG5	6q14.3	387066	Oncogene/antioncogene
SNHG6	8q13.1	641638	Oncogene
SNHG7	9q34.3	84973	Oncogene/antioncogene
SNHG8	4q26	100093630	Oncogene
SNHG9	16p13.3	735301	Oncogene/antioncogene
SNHG10	14q32.13	283596	Antioncogene
SNHG11	20q11.23	128439	Oncogene
SNHG12	1p35.3	85028	Oncogene
DANCR	4q12	57291	Oncogene
SNHG14	15q11.2	104472715	Oncogene
SNHG15	7p13	285958	Oncogene
SNHG16	17q25.1	100507246	Oncogene
SNHG17	20q11.23	388796	Oncogene
SNHG18	5p15.31	100505806	Oncogene
SNHG20	17q25.2	654434	Oncogene

SNHG: small nucleolar RNA host gene.

### 2.2 LncRNA

SNHGs与NSCLC的诊断及预后 随着高分辨率计算机断层扫描（computed tomography, CT）在临床的普及，胸部CT已成为筛查早期NSCLC的重要手段。早期NSCLC在胸部CT上表现为含有磨玻璃成分的部分实性结节。胸外科医生可根据CT上结节的特征，如肿块的大小，有无分叶、毛刺，有无胸膜牵拉征、血管集束征等，对结节的良恶性进行评估。然而，由于部分CT软硬件的局限性导致的图像不清、图层过厚，使得肿瘤的筛查结果容易出现假阴性，且不同的研究中心、不同的医生对结节的认定也存在偏差。因此，需要开发更灵敏精确的技术手段用于NSCLC的早期诊断及预后评估，而新型生物标志物的探索受到了学者们的广泛关注。

近年来，大量研究证实了lncRNA SNHGs对NSCLC诊断及预后的潜在价值。Mourgi等^[31]^分析了100例NSCLC患者手术前后血浆中GAS5的表达，发现GAS5在NSCLC的组织和血浆中表达较低，但在手术后却显著升高。Kaplan-Meier曲线生存分析显示，GAS5的表达下调与NSCLC患者的不良预后相关。Li所在团队^[32]^为评估GAS5在外泌体（Exo-GAS5）中的表达和诊断价值，入组了104例受试者，应用外泌体分离试剂盒提取RNA并检测表达量。受试者工作特征（receiver operating characteristic, ROC）曲线分析结果显示Exo-GAS5可用于鉴别I期NSCLC患者，与肿瘤大小、肿瘤原发灶-淋巴结-转移（tumor-node-metastasis, TNM）分期直接相关，是识别早期NSCLC患者理想的无创性血清标志物。此外，最新的一项研究^[33]^发现，GAS5的单核苷酸多态性（single nucleotide polymorphism, SNP）rs145204276变异可能有助于预测表皮生长因子受体（epidermal growth factor receptor, EGFR）野生型肺腺癌患者的肿瘤分期、淋巴结转移和远处转移。Wang等^[23]^研究了50例NSCLC患者的组织样本，发现SNHG4在肿瘤组织中高表达，Kaplan-Meier生存分析曲线揭示了lncRNA SNHG4与NSCLC患者预后之间的显著相关性。2018年，Liang所在团队^[34]^首次检测出SNHG6在NSCLC组织中上调，并且与NSCLC患者中的晚期TNM分期、肿瘤较大和较差的总生存期（overall survival, OS）呈正相关。同年，Chen所在团队^[26]^分析了120例NSCLC患者的组织样本，发现SNHG8在NSCLC中过表达，且SNHG8高表达患者的OS和无进展生存期（progression-free survival, PFS）远低于SNHG8低表达患者。Cui等^[35]^通过qRT-PCR检测了55例NSCLC组织和30例正常肺组织中SNHG15的表达量，并应用Kaplan-Meier法分析SNHG15的表达与NSCLC病理特征的关系，结果显示，SNHG15在NSCLC组织中的表达较高，且SNHG15的表达与NSCLC患者的肿瘤大小、TNM分期和淋巴结转移有关。Han所在团队^[30]^研究发现，较正常组织相比，SNHG16在NSCLC组织中表达上调。Kaplan-Meier分析和多因素Cox回归分析显示，SNHG16的表达量可作为NSCLC患者生存预后的独立预测因子。

总之，多种lncRNA SNHGs在NSCLC组织中的表达量与肿瘤的临床分期及患者的预后高度相关，为NSCLC的早期筛查、临床分期和预后分析提供了新的思路和方向。然而，样本量的不足使得目前的研究仍存在一定局限性，且检测方法的选择、SNHGs作为诊断和预后评估的灵敏度和特异度如何、样本的入选标准等问题也亟待解决。

## 3 LncRNA SNHGs的致癌机制

### 3.1 ceRNA机制

2011年，Salmena等^[36]^首次提出了ceRNA假说，开启了一扇转录组研究的新大门。该假说认为，细胞内不同的RNA分子（lncRNA、mRNA、circRNA等）能够通过miRNA应答元件（microRNA response element, MRE）竞争结合相同的miRNA来调节彼此的表达水平。MRE是miRNA与靶RNA转录物上部分互补性的序列。miRNA通过MRE与mRNA结合，导致mRNA降解或者抑制其翻译，从而负性调控基因的表达。而当细胞内的lncRNA与mRNA有相同的MRE时，他们之间构成了竞争相同种类miRNA的关系。因此，lncRNA表达水平的高低，直接决定了可被相应mRNA结合的miRNA数量的多少，即改变了mRNA的表达量，进而影响肿瘤的生物学特性。

在NSCLC中，lncRNA SNHGs通过充当ceRNA参与肿瘤的功能调控。例如，SNHG1通过结合miR-101-3p，靶向作用于SRY-box转录因子9（SRY-box transcription factor 9, SOX9），并激活Wnt/β-catenin信号通路促进NSCLC细胞的增殖、侵袭和转移能力^[11]^。Li等^[37]^发现，SNHG1可与miR-497结合并抑制其表达，导致胰岛素样生长因子1受体（insulin-like growth factor 1 receptor, IGF1-R）的表达升高，促进NSCLC的生物学进展。此外，SNHG1通过充当miR-145-5p的分子海绵，上调癌基因MTDH的表达，加速肿瘤的发生发展^[38]^。SNHG1还可以结合miR-361-3p并调控FRAT1（frequently rearranged in advanced T-cell lymphomas 1）表达，进而在NSCLC中起到促癌作用^[39]^。Ma所在团队^[40]^发现GAS5通过抑制miR-221-3p增加干扰素调节因子2（interferon regulatory factor 2, IRF2）表达水平，从而延缓NSCLC的进展。Zhao等^[41]^提出，SNHG3通过干扰miR-216a来增强锌指E-box结合同源盒1（zinc finger E-box binding homeobox 1, ZEB1）的表达，促进了NSCLC肿瘤的发生和发展。Li等^[42]^的研究发现，SNHG3可以靶向作用于miR-515-5p，并正向调节小泛素样修饰物2（small ubiquitin like modifier 2, SUMO2），从而影响NSCLC细胞的增殖和转移。另有研究^[43]^表明，SNHG3/miR-1343-3p/核因子IX（nuclear factor IX, NFIX）轴可能成为NSCLC的预后生物标志物和治疗靶点。一项研究^[44]^发现，SNHG5在NSCLC组织中表达量上调，并通过调控miR-181c-5p/chromobox蛋白4（chromobox protein 4, CBX4）轴诱导NF-κB通路促进NSCLC进展。Li所在团队^[45]^指出，SNHG6通过下调miR-101-3p并作用于CDYL（chromodomain Y like），促进NSCLC的增殖和侵袭能力。另一项研究^[46]^发现，SNHG6可以靶向作用于miR-490-3p，调节重构和间隔因子1（remodeling and spacing factor 1, RSF1）的表达，促进体内肿瘤生长。此外，SNHG6还通过miR-944和miR-181d-5p调控ETS原癌基因1（ETS proto-oncogene 1, ETS1）信号通路参与NSCLC的进展^[47]^。Pei所在团队^[48]^指出，SNHG7与miR-181相互作用并顺序上调CBX7（chromobox 7），而CBX7抑制NSCLC中的Wnt/β-catenin通路从而发挥生物学效应。随后的一项研究^[49]^显示，SNHG7充当miR-181a-5p的分子海绵，并靶向作用于E2F7，正向促进NSCLC的发生发展。Zhang等^[50]^的研究发现，SNHG10通过结合miR-543上调NSCLC中的沉默调节蛋白1（sirtuin 1, SIRT1）以抑制肿瘤增殖、迁移和侵袭。Liu等^[51]^发现SNHG11不仅可以靶向作用于miR-4436 a/CTNNB1（catenin beta 1）轴来激活Wnt/β-catenin信号通路发挥生物学效应，还能够直接与β-catenin结合激活经典Wnt通路，促进肿瘤进展。随后，Cheng所在团队^[52]^提出，SNHG11充当了miR-485-5p的分子海绵，并直接靶向BSG（basigin）发挥调控功能。此外，SNHG11还可以调控miR-193a-5p/Notch3轴激活Notch通路，促进NSCLC的增殖和迁移^[53]^。Xie等^[54]^发现SNHG12通过结合miR-101-3p调节CUL4B（cullin 4B）介导的PI3K/AKT通路，发挥生物学效应。Bai所在团队^[55]^发现，DANCR可以和SRY-box转录因子4（SRY-box transcription factor 4, SOX4）竞争性结合miR-138，从而影响SOX4的表达，促进NSCLC的生长和转移。此外，DANCR还通过调控miR-216a/Wnt/β-catenin轴加速肿瘤进展^[56]^，因此，DANCR可能成为NSCLC新的治疗靶标。一项研究^[57]^表明，SNHG14充当了miR-382-5p的分子海绵，调控SPIN1的表达，促进NSCLC的进展。此外，SNHG14还通过miR-206/葡萄糖-6-磷酸脱氢酶（glucose-6-phosphate dehydrogenase, G6PD）轴参与NSCLC的发生发展^[58]^，为人们了解NSCLC的发病机制提供了新的依据。Chen等的研究^[59]^表明，SNHG16可以作为ceRNA调节miR-520/血管内皮生长因子（vascular endothelial growth factor, VEGF）轴并促进NSCLC细胞的迀移。Yu所在团队^[60]^提出，SNHG16通过作为miR-520a-3p的分子海绵，减少其抑制EPH受体A2（EPH receptor A2, EphA2）的能力，进而促进NSCLC的发生发展。研究^[61]^发现，SNHG17可以抑制miR-193a-5p的表达并靶向调控NETO2（neuropilin and tolloid like 2）的水平，从而促进NSCLC细胞的增殖、迁移和侵袭。SNHG17还可以作用于miR-485-5p并上调Wnt配体分泌介质（Wnt ligand secretion mediator, WLS）的表达，加速肿瘤的进展^[62]^。Fan等^[63]^的研究表明，SNHG18通过调节miR-211-5p/BRD4轴促进NSCLC的生长和转移。Wang所在团队发现SNHG20的敲低通过结合miR-342靶向调控DEAD-box解旋酶49（DEAD-box helicase 49, DDX49）的表达，抑制肿瘤的进展^[64]^。SNHG20还通过海绵化miR-154来上调锌指E-box结合同源盒2（zinc finger E-box binding homeobox 2, ZEB2）和Runt-相关转录因子2（runt related transcription factor 2, RUNX2）表达，促进细胞凋亡^[65]^。Chen等^[66]^指出，SNHG20可以结合miR-2467-3p，激活AKT信号通路，并增加NSCLC细胞中的E2F转录因子3（E2F transcription factor 3, E2F3）水平来发挥效应。SNHG20的下调可以负调节miR-197的表达，并通过Wnt/β-catenin信号通路促进NSCLC的生物学进展^[67]^。

近年来，越来越多的研究探索了NSCLC中的ceRNA机制，使得lncRNA SNHGs在NSCLC中的ceRNA调控网络越来越完善。然而学者们在实验中对ceRNA相互作用的演示往往局限于单个基因，但在细胞中，一个lncRNA可以充当多个miRNA的ceRNA，而一个mRNA又可被多个miRNA识别，这使得对ceRNA调控网络的追根溯源成为一大难题。

### 3.2 其他机制研究

除了研究最多的ceRNA机制，LncRNA SNHGs还能通过与转录因子相互作用或直接结合mRNA等途径参与肿瘤进展的调控。例如，SNHG1可以与EGFR结合在NSCLC进展中发挥作用^[19]^。Zhu所在团队^[21]^发现，GAS5通过增加E-cadherin并减少N-cadherin的表达参与调控EMT通路，显著抑制NSCLC细胞的侵袭和转移能力。GAS5还可以与UPF1相互作用促进NSCLC细胞的自噬活性，延缓肿瘤的发生发展^[22]^。Shi所在团队^[68]^发现，SNHG3是E2F转录因子1（E2F transcription factor 1, E2F1）的直接转录靶标，E2F1通过转化生长因子β（transforming growth factor β, TGF-β）通路和IL-6/JAK2/STAT3通路激活SNHG3，促进NSCLC细胞增殖和迁移，提示SNHG3可能是NSCLC患者治疗的生物标志物。Kang所在团队^[69]^的实验结果显示，SNHG3还通过调控miR-890的表达促进NSCLC的发生和发展。另一项研究^[70]^指出，SNHG7通过磷酸激酶B（protein kinase B, AKT）/哺乳动物雷帕霉素靶蛋白（mammalian target of rapamycin, mTOR）信号通路抑制miR-181a-5p，促进NSCLC的增殖、迁移和侵袭。Chai等^[71]^研究发现，槲皮素可以降低NSCLC中SNHG7表达，并上调miR-34a-5p，抑制肿瘤细胞增殖并诱导细胞凋亡。Wang所在团队^[72]^对癌症基因组图集（The Cancer Genome Atlas, TCGA）数据库的分析及64组NSCLC患者样本的检测表明，SNHG9在NSCLC组织中呈现低表达，并通过甲基化下调miR-21的水平，抑制肿瘤细胞的增殖。Liang等^[73]^在研究中指出，SNHG10也可以使miR-21基因甲基化而降低其表达，延缓NSCLC的进展。一项研究^[74]^表明，SNHG12通过抑制miR-218的表达水平，并促进Slug/ZEB2信号通路，从而诱导细胞的迁移和侵袭。SNHG12还可以结合miR-138，促进肿瘤细胞的增殖和转移^[75]^。此外，SNHG12与人抗原R（human antigen R, HuR）的结合改善了程序性细胞死亡配体1（programmed cell death ligand 1, PD-L1）和泛素特异性蛋白酶8（ubiquitin-specific protease 8, USP8）的mRNA稳定性和表达，而USP8介导的去泛素化稳定了PD-L1的蛋白质水平，由此促进了NSCLC的免疫逃逸，加速了NSCLC的进程^[27]^。Li所在团队^[76]^指出，SNHG16可以与乙醛脱氢酶2（aldehyde dehydrogenase 2, ALDH2）相互作用而发挥功能。另有研究^[77]^表明，SNHG20与EZH2的增强子结合后对p21的调控减弱，进而诱导NSCLC细胞迁移和增殖，并抑制细胞凋亡。

总之，lncRNA SNHGs在NSCLC中的调控机制错综复杂，未来仍需更多更深入的研究来充分实现其临床价值。

## 4 LncRNA SNHGs与药物抗性的机制研究

NSCLC的治疗手段主要有手术、放疗、化疗、靶向治疗、免疫治疗等。随着电视辅助胸腔镜技术的成熟与普及，外科手术逐渐成为了早期NSCLC的首选治疗。然而许多患者术后仍可能发生局部复发或远处转移。近年来，化疗和分子靶向治疗等辅助治疗领域的突破与进展为这类NSCLC患者带来福音，大幅提高了患者的存活率和生活质量。但随之而来的获得性耐药却又使得晚期NSCLC的治疗困难重重。

研究^[78⇓⇓-81]^表明，lncRNA SNHGs会导致NSCLC细胞对顺铂等化疗药物产生耐药性。肿瘤细胞对顺铂产生耐药主要归因于顺铂-DNA加合物的耐受性和DNA损伤的修复，此外还与抗凋亡信号的诱导、药物从细胞质主动外排、miRNA的表观遗传调节、免疫系统的抑制等因素相关。Ge所在团队^[82]^的研究发现SNHG1在对顺铂耐药的NSCLC组织和细胞中表达显著上调，随后他们提出并证明SNHG1通过靶向miR-330-5p上调双皮质素样激酶1（doublecortin-like kinase 1, DCLK1）的表达，提高了NSCLC细胞对顺铂的耐药性，促进了肿瘤的恶性生物学行为。另有研究^[83]^报道GAS5可以通过miR-217/磷赖氨酸磷酸组氨酸无机焦磷酸磷酸酶（phospholysine phosphohistidine inorganic pyrophosphate phosphatase, LHPP）轴降低NSCLC的顺铂耐药性。肿瘤耐药已被证实与耐药基因多药耐药蛋白1（multidrug resistance protein 1, MDR1）^[84]^、乳癌耐性蛋白（breast cancer resistance protein, BCRP）^[85]^和磷脂酰肌醇-3-激酶（phosphatidylinositol-3-hydroxykinase, PI3K）/AKT/mTOR^[86]^通路有关。Chen等^[87]^使用qRT-PCR检测发现，敲降SNHG7后，PI3K/AKT通路中的耐药基因及相关基因的表达显著下调，进一步的功能实验证明SNHG7通过PI3K/AKT通路上调MRD1和BCRP，诱导NSCLC细胞对顺铂耐药。另一篇文章^[88]^指出，SNHG7可以上调肿瘤细胞中的自噬相关蛋白，并抑制P62的表达，诱导自噬活性而促进NSCLC细胞的恶性行为和顺铂抗性。Zhang所在团队^[89]^发现，外泌体SNHG7通过募集HuR稳定自噬相关基因5（autophagy-related genes autophagy related 5, ATG5）和自噬相关基因12（autophagy-related genes autophagy related 12, ATG12）来促进自噬，并激活PI3K/AKT途径导致巨噬细胞中的M2极化，二者共同调控了NSCLC细胞对多西他赛的抗性。此外，SNHG9通过正向调控CAPRIN 1的表达诱导NSCLC细胞对顺铂产生耐药性^[90]^。一项研究^[91]^发现，SNHG12能抑制miR-525-5p的表达并促进X连锁细胞凋亡抑制剂（X-linked inhibitor of apoptosis, XIAP）的转录，增强NSCLC细胞的顺铂耐药性。Tan等^[92]^在评估SNHG12在癌相关成纤维细胞（carcinoma-associated fibroblasts, CAFs）起源的细胞外囊泡（extracellular vesicles, EV）中对NSCLC细胞顺铂耐药的调节作用时发现，由CAFs-EV携带到肿瘤细胞中的SNHG12通过与HuR结合而促进RNA稳定性和XIAP转录，从而增强NSCLC细胞中的顺铂抗性。Jiao等^[93]^的机制研究结果显示，SNHG14通过miR-34a/高迁移率族蛋白1（high mobility group box 1, HMGB1）轴促进NSCLC进展，并提高NSCLC细胞对顺铂的耐药性，提示SNHG14可能是NSCLC治疗的潜在靶点。Li所在团队^[94]^提出，SNHG15可以募集E2F转录因子2（E2F transcription factor 2, E2F2）来上调内皮素转化酶1（endothelin converting enzyme 1, ECE1）表达，从而增强NSCLC对顺铂的耐药性。

此外，lncRNA SNHGs还参与了NSCLC细胞对吉非替尼等分子靶向药物的耐药过程。吉非替尼属于EGFR-酪氨酸激酶抑制剂（EGFR-tyrosine kinase inhibitors, EGFR-TKIs），其常见的耐药机制有EGFR T790M基因突变、MET基因扩增、PIK3CA基因突变，以及转化为小细胞肺癌等^[95⇓-97]^。有研究^[98]^提示，SNHG5可以通过结合miR-377靶向作用于含半胱氨酸的天冬氨酸蛋白水解酶1（cysteinyl aspartate specific proteinase 1, CASP1），从而提高NSCLC细胞对吉非替尼靶向治疗的耐受性。2014年，Wang所在团队^[99]^在探索NSCLC多重耐药的相关机制时，首次发现SNHG12在NSCLC肿瘤组织和细胞系中高表达。功能实验表明，沉默SNHG12可通过miR-181a调节MAPK/Slug通路发挥作用，诱导细胞凋亡，逆转肿瘤细胞对顺铂、紫杉醇和吉非替尼的耐药性。Wu等^[100]^的研究指出，SNHG14可以调节miR-206-3p/ABCB1（ATP binding cassette subfamily B member 1）通路，进而增强肿瘤细胞对吉非替尼的耐药性。NOTCH通路与NSCLC的化学抗性相关，当NSCLC细胞中NOTCH-1耗尽后，SNHG15表达升高。功能研究^[101]^表明，SNHG15和MDR1在吉非替尼耐药的肺癌细胞中过表达并具有促肿瘤作用，而SNHG15可以通过调节miR-451靶向作用于MDR1，从而改变NSCLC细胞对吉非替尼的耐药性。Zhang所在团队^[102]^研究发现，甲基转移酶3（methyltransferase-like 3, METTL3）介导的N6-甲基腺苷修饰可以通过稳定其RNA转录物来诱导SNHG17的上调，降低了NSCLC细胞对吉非替尼等药物的敏感性。随后的功能研究结果显示，SNHG17通过将EZH2募集到大肿瘤抑制激酶2（large tumor suppressor kinase 2, LATS2）的启动子区域，进而抑制了LATS2的表达，使肿瘤细胞产生耐药性。

然而，lncRNA SNHGs在肿瘤细胞耐药中的机制研究仍需进一步探索，为新型靶向药物的问世提供更多的灵感和思路。

## 5 总结与展望

随着单细胞测序技术的广泛应用，越来越多的lncRNA得以被发现，人们逐渐认识到lncRNA在表观遗传学和肿瘤进展中的重要作用。随着研究的深入，lncRNA在恶性肿瘤中的异常表达和调控机制被逐步揭示。SNHGs作为一类新发现的lncRNA，其表达水平与肿瘤的病理生理特征及患者预后存在着紧密关联。本文总结并阐述了lncRNA SNHGs的异常表达与NSCLC的预后（如TNM分期、淋巴结转移、OS）和功能（如增殖、侵袭、迁移、凋亡、自噬和化疗耐药）之间的密切联系及相关分子机制。在机制方面，SNHGs可以通过结合转录因子启动转录、直接作用并调节mRNA、作为ceRNA吸附各种miRNA、激活各种信号通路等参与调节癌症的恶性生物学行为，不同程度地促进NSCLC细胞的增殖、侵袭、迁移，干扰NSCLC细胞的凋亡和自噬，显著影响NSCLC患者的预后（表2^[11,19⇓⇓⇓⇓⇓⇓⇓⇓⇓⇓-30,32⇓⇓-35,37⇓⇓⇓⇓⇓⇓⇓⇓⇓⇓⇓⇓⇓⇓⇓⇓⇓⇓⇓⇓⇓⇓⇓⇓⇓⇓⇓⇓⇓⇓⇓⇓⇓⇓⇓⇓⇓⇓⇓-77,82,83,87⇓⇓⇓⇓⇓⇓-94,98⇓⇓⇓-102]^）。

**表2:** SNHG家族成员在非小细胞肺癌中的研究进展

SNHG member	In vivo	Mechanism	Related signaling pathway	Drug resistance	Ref
SNHG1	√	SNHG1/miR-101-3p/SOX9; SNHG1/miR-145-5p/MTDH; SNHG1/miR-361-3p/FRAT1; SNHG1/miR-497/IGF1-R; SNHG1/miR-330-5p/DCLK1; SNHG1/EGFR	Wnt/β-catenin; NF-κB; STAT1	Cisplatin	^[11,19,37⇓-39,82]^
GAS5	√	GAS5/miR-217/LHPP; GAS5/miR 221-3p/IRF2; GAS5/ miR-221-3p/TP63; GAS5/Ki-67, EGFR, E-cadherin, N-cadherin; GAS5/UPF1/BRD4		Cisplatin	^[20⇓-22,32,33,40,83]^
SNHG3		SNHG3/miR-515-5p/SUMO2; SNHG3/miR-890; SNHG3/miR-1343-3p/NFIX; SNHG3/miR-216a/ZEB1; E2F1/SNHG3	TGF-β; IL-6/JAK2/STAT3		^[41⇓-43,68,69]^
SNHG4		SNHG4/let-7e/KDM3A/p21			^[23]^
SNHG5		SNHG5/miR-377/CASP1; SNHG/miR-181c-5p/CBX4; SNHG5/X-linked inhibitor of apoptosis protein		TRAIL; Gefitinib	^[44,98]^
SNHG6	√	SNHG6/miR-101-3p; SNHG6/miR-26a-5p/E2F7; SNHG6/miR-490-3p/RSF1; SNHG6/miR-944, miR-181d-5p/ETS1; SNHG6/p27			^[24,25,34,45⇓-47]^
SNHG7	√	SNHG7/miR-34a-5p; SNHG7/miR-181a-5p/E2F7; SNHG7/miR-181/cbx7; SNHG7/miR-181a-5p; SNHG7/MRD1, BCRP	AKT/mTOR; PI3K/AKT; Wnt/β-catenin	Cisplatin; Docetaxel	^[48,49,70,71,87⇓ -89]^
SNHG8		SNHG8/miR-542-3p/CCND1; CDK6			^[26]^
SNHG9		SNHG9/miR-21; SNHG9/CAPRIN 1		Cisplatin	^[72,90]^
SNHG10		SNHG10/miR-21; SNHG10/miR-543/SIRT1;			^[50,73]^
SNHG11		SNHG11/miR-485-5p/BSG; SNHG11/miR-4436a/CTNNB1; SNHG11/miR-193a-5p/Notch3	Wnt/β-catenin; Notch		^[51⇓-53]^
SNHG12	√	SNHG12/HuR/XIAP; SNHG12/miR-218; SNHG12/miR-181a; SNHG12/miR-138; SNHG12/miR 101-3p/CUL4B; SNHG12/miR-525-5p/XIAP; SNHG12/HuR/PD-L1, USP8	Slug/ZEB2; MAPK/Slug; PI3K/AKT	Cisplatin	^[27,54,74,75,91,92,99]^
DANCR		DANCR/miR-216a; DANCR/miR-138/Sox4; DANCR/p21	Wnt/β-catenin		^[28,55,56]^
SNHG14	√	SNHG14/miR-206/G6PD; SNHG14/miR-206-3p/ABCB1; SNHG14/miR-382-5p/SPIN1; SNHG14/miR-34a/HMGB1		Gefitinib; Cisplatin	^[57,58,93,100]^
SNHG15	√	SNHG15/miR-486/CDK14; SNHG15/miR-211-3p; SNHG15/miR-451/MDR1; SNHG15/ECE1	Notch	Gefitinib; Cisplatin	^[29,35,94,101]^
SNHG16	√	SNHG16/miR-520/VEGF; SNHG16/miR-146a/MUC5AC; SNHG16/ALDH 2; SNHG16/miR 520a-3p/EphA2			^[30,59,60,76]^
SNHG17		SNHG17/miR-193a-5p/NETO2; SNHG17/miR-485-5p/WLS; METTL3/SNHG17/LATS2		Gefitinib	^[61,62,102]^
SNHG18	√	SNHG18/miR-211-5p/BRD4			^[63]^
SNHG20		SNHG20/miR-342/DDX49; SNHG20/miR-2467-3p/E2F3; SNHG20/miR-154/ZEB2, RUNX2; SNHG20/miR-197; SNHG20/p21	Wnt/β-catenin; AKT		^[64⇓⇓-67,77]^

SOX9: SRY-box transcription factor 9; IGF1-R: insulin-like growth factor 1 receptor; MTDH: metadherin; FRAT1: frequently rearranged in advanced T-cell lymphomas 1; DCLK1: doublecortin-like kinase 1; IL: interleukin; EGFR: epidermal growth factor receptor; IRF2: interferon regulatory factor 2; TP63: Tumor protein p63; LHPP: phospholysine phosphohistidine inorganic pyrophosphate phosphatase; FTO: fat mass and obesity-associated protein; UPF1: up-frameshift protein 1; BRD4: bromodomain-containing protein 4; E2F1: E2F transcription factor 1; TGF-β: transforming growth factor-β; ZEB1: zinc finger E-box binding homeobox 1; SUMO2: small ubiquitin like modifier 2; NFIX: nuclear factor IX; KDM3A: lysine demethylase 3A; CBX4: chromobox protein 4; CASP1: cysteinyl aspartate specific proteinase 1; TRAIL: TNF-related apoptosis-inducing ligand; E2F7: E2F transcription factor 7; CDYL: chromodomain Y like; RSF1: remodeling and spacing factor 1; ETS1: ETS proto-oncogene 1; EZH2: enhancer of zeste 2 polycomb repressive complex 2 subunit; CBX7: chromobox 7; MDR-1: multidrug resistance protein 1; BCRP: breast cancer resistance protein; ATG5: autophagy-related genes autophagy related 5; ATG12: autophagy-related genes autophagy related 12; CCND1: cyclin D1; CDK6: cyclin dependent kinase 6; SIRT1: sirtuin 1; CTNNB1: catenin beta 1; BSG: basigin; CUL4B: cullin 4B; XIAP: X-linked inhibitor of apoptosis; ZEB2: zinc finger E-box binding homeobox 2; HuR: human antigen R; PD-L1: programmed cell death receptor ligand 1; USP8: ubiquitin-specific protease 8; SOX4: SRY-box transcription factor 4; HMGB1: high mobility group box 1; ABCB1: ATP binding cassette subfamily B member 1; SPIN1: spindlin 1; G6PD: glucose-6-phosphate dehydrogenase; CDK14: cyclin dependent kinase 14; E2F2: E2F transcription factor 2; ECE1: endothelin converting enzyme 1; MUC5AC: mucin 5AC; EphA2: EPH receptor A2; ALDH2: aldehyde dehydrogenase 2; NETO2: neuropilin and tolloid like 2; WLS: Wnt ligand secretion mediator; METTL3: methyltransferase-like 3; LATS2: large tumor suppressor kinase 2; MKL1: megakaryocytic leukemia 1; DDX49: DEAD-box helicase 49; E2F3: E2F transcription factor 3; RUNX2: runt related transcription factor 2.

在过去的10年中，靶向治疗和免疫治疗的出现为NSCLC患者带来福音，无论是单独使用还是与化疗联合，它们都已广泛应用于临床一线。尽管如今靶向治疗和免疫治疗都已取得了重大突破，但NSCLC患者的总体PFS仍然很低。为了给每位患者定制合适的靶向治疗方案，在分子水平上评估其特定的敏感基因及耐药机制变得极为重要，毫无疑问lncRNA SNHGs在这中间发挥着举足轻重的作用。相信在未来，随着空间多组学技术的日益成熟及新型靶向材料的问世，lncRNA SNHGs在NSCLC中的作用机制及应用价值必将被进一步挖掘，为NSCLC的早期诊断及预后评估、识别新型生物靶标、逆转肿瘤多药耐药等开辟新的领域。

**Competing interests**

The authors declare that they have no competing interests.
